# Capacity Planning for Batch and Perfusion Bioprocesses Across Multiple Biopharmaceutical Facilities

**DOI:** 10.1002/btpr.1860

**Published:** 2014-01-24

**Authors:** Cyrus C Siganporia, Soumitra Ghosh, Thomas Daszkowski, Lazaros G Papageorgiou, Suzanne S Farid

**Affiliations:** 1 Dept. of Biochemical Engineering, University College LondonLondon, WC1E 7JE, U.K.; 2 Biotechnology Controlling, Bayer HealthcareBerkeley, California, USA; 3 Dept. of Chemical Engineering, Centre for Process Systems Engineering, University College LondonLondon WC1E 7JE, U.K.; 4 Process Development and Optimisation, Bayer AGLeverkusen, Germany; 5 Dept. of Biochemical Engineering, University College LondonLondon, WC1E 7JE, U.K.

**Keywords:** capacity planning, scheduling, business decision-making, mixed integer linear programming, rolling time horizon

## Abstract

Production planning for biopharmaceutical portfolios becomes more complex when products switch between fed-batch and continuous perfusion culture processes. This article describes the development of a discrete-time mixed integer linear programming (MILP) model to optimize capacity plans for multiple biopharmaceutical products, with either batch or perfusion bioprocesses, across multiple facilities to meet quarterly demands. The model comprised specific features to account for products with fed-batch or perfusion culture processes such as sequence-dependent changeover times, continuous culture constraints, and decoupled upstream and downstream operations that permit independent scheduling of each. Strategic inventory levels were accounted for by applying cost penalties when they were not met. A rolling time horizon methodology was utilized in conjunction with the MILP model and was shown to obtain solutions with greater optimality in less computational time than the full-scale model. The model was applied to an industrial case study to illustrate how the framework aids decisions regarding outsourcing capacity to third party manufacturers or building new facilities. The impact of variations on key parameters such as demand or titres on the optimal production plans and costs was captured. The analysis identified the critical ratio of in-house to contract manufacturing organization (CMO) manufacturing costs that led the optimization results to favor building a future facility over using a CMO. The tool predicted that if titres were higher than expected then the optimal solution would allocate more production to in-house facilities, where manufacturing costs were lower. Utilization graphs indicated when capacity expansion should be considered. © 2013 The Authors Biotechnology Progress published by Wiley Periodicals, Inc. on behalf of American Institute of Chemical Engineers *Biotechnol. Prog*., 30:594–606, 2014

## Introduction

Biopharmaceutical companies with growing portfolios of commercial therapeutics face the challenge of generating medium- and long-term production plans for several drugs across several multiproduct manufacturing sites that maximize capacity whilst minimizing cost. There are various examples of the repercussions of incorrect capacity planning, including high-profile company acquisitions owing to over- and under-capacity, respectively.^1^ Capacity sourcing strategies for biopharmaceutical companies often involve consideration of build-vs.-buy decisions, that is, choosing whether to build in-house facilities or outsource manufacturing to a contract manufacturing organization (CMO).^2^ Developing a comprehensive production planning strategy requires careful assessment of the cost, risk, and time trade-offs of each option.^1^,^3^,^4^

Decisions to build a facility for commercial production need to be scheduled several years in advance before a drug's full market potential, likely dose range, cell line productivity, and process yields are known. The use of CMOs enables such capital outlays to be delayed whilst incurring a premium for their services. A further factor affecting the decision relates to the relative difference in manufacturing efficiencies assumed between in-house and external manufacturing. In the case study presented in this article, third party manufacturers were assumed to have higher manufacturing yields than the drug developer company.^5^

By outsourcing to CMOs, biopharmaceutical companies can mitigate risks concerning failed batches, natural disasters, incorrect market demand forecasts, or a clinical trial failure. The downside of using CMOs is usually the loss of process control, or delays in technology transfer to in-house facilities if later required.^6^ Building a new facility conversely, requires consideration of the lead time for construction, commissioning, and validation of the facility, all of which can take up to 4 years to complete, and can cost $40–650M for large commercial antibody facilities.^7^ Consequently, more effective optimization frameworks are required to facilitate capacity sourcing decisions across a network of existing in-house and CMO facilities as well as potential new builds so as to ensure the availability of sufficient capacity whilst minimising running costs and capital outlays.

Production planning is complicated by portfolios of commercial candidates that are made with different cell culture modes: fed-batch mode, or continuous perfusion for labile products. The complication arises from the fact that perfusion cell cultures can span many months, whereas fed-batch cell cultures are usually 2 weeks in duration. The discrete time representation used in this model is of 1 month, thus extra modeling constraints need to be introduced to ensure that production is not stopped half-way through a cell culture (as it is meant to model a continuous process). Every time a new perfusion cell culture begins, ramp-up times need to be considered as the manufacturer may choose not to harvest any material during this period, as it is not meeting all required specifications. Material is normally harvested semicontinuously from perfusion processes, which is conceptually different from a fed-batch process where material is harvested at the end of the cell culture. Thus, these continuous harvests need to be incorporated in the model's constraints. Changing from one mode of operation to another also increases the changeover time normally associated with product switchovers. This adds more complexity to the optimization as a larger number of constraints are required.

Previous work on production planning has been limited to batch processes and has revolved predominantly around mathematical programming, most commonly mixed integer linear programming (MILP). Early research into capacity planning^8^–^11^ produced general mathematical formulations for multiple campaigns in multipurpose batch plants with features such as campaign changeovers and inventory profiles. These included models for the pharmaceutical sector using a mathematical MILP formulation that addressed strategies in capacity planning, product development, and investment where taxation and different sales regions were captured.^12^ The model used time periods of 1 year, and hence it was solely to be used for capacity planning rather than scheduling. Lakdhar et al.^13^ developed an MILP for the planning and scheduling of a multi-product biopharmaceutical manufacturing facility and showed it to be more efficient in terms of facility utilization and cost reduction than the standard industrial rule-based approach. This model was later expanded into a multi-facility and multi-product model, where fluctuations in demand were considered, as well as multi-objective criteria such as customer service level and facility utilization by means of goal programming.^3^

Short-term scheduling of batch plants with sequence-dependent changeover times has been addressed using continuous-time representation MILP models with either binary variables or extra constraints.^14^ Combined planning and scheduling models can be computationally expensive and have been tackled by different approaches such as a multi-stage MILP approach^15^ and mathematical programming formulations with separate scheduling and planning aspects of supply chain optimization, which are then linked sequentially via a common time basis.^16^

Biological systems often show great variability in productivity during early development, and thus attempts to capture uncertainties within the model are important. For pharmaceutical (non-biologic) products, capacity planning under uncertainty in clinical trials has been addressed using an MILP model with a hierarchal procedure to improve performance of large models,^17^ a scenario-based aggregation/disaggregation procedure^18^ and a framework, which includes both stochastic simulation and an MILP model.^19^ For biopharmaceutical products, both evolutionary algorithms and mathematical programming approaches have been developed. Lakhdar et al. captured the impact of uncertain fermentation titres on medium-term production plans of biopharmaceutical products using chance-constrained programming.^20^ George and Farid addressed capacity planning under several clinical, technical, and commercial uncertainties using stochastic evolutionary algorithms.^21^

Most biopharmaceutical production planning work^20^,^21^ has focused on batch rather than continuous processes. Perfusion mode is not as common as batch mode, but in certain circumstances (e.g., when product stability is an issue) it is the only option available to manufacturers. In these cases, a framework which can help them predict capacity bottlenecks and manufacturing profitability would aid decision-making on key questions such as whether to outsource production or not.

This article describes the development of a discrete-time MILP model that incorporates both perfusion and fed-batch processes to produce capacity plans and manufacturing schedules. Extra constraints have been incorporated to more realistically model the perfusion process. For example, ramp-up times and cell culture durations spanning multiple time periods have been implemented for perfusion-mode processes. One of the challenges met by this formulation is the ability to include sequence-dependent changeover times between products, which is necessary because switching between perfusion and fed-batch modes takes longer than staying within the same process mode. Annual fixed costs are also included in the model, along with other investment considerations such as retrofitting costs and investment into constructing new facilities. These additional features allow the model to pick strategies based on a more holistic approach, and thus provide more economically feasible solutions. Strategic inventory targets have also been implemented such that the manufacturer can choose to have extra stock of product should demand unexpectedly rise or supply suddenly fall. These extra features add to the complexity of the model, and thus require additional central processing unit (CPU) resources to obtain a satisfactory solution. Hence, a rolling time horizon has been implemented and has successfully managed to improve solutions, whilst at the same time reduce time requirements. The impact of variations on key parameters such as demand or titres on the optimal production plans and costs was captured through scenario analysis.

The remainder of this article consists of an explanation of the problem domain in Section “Problem Definition,” followed by a description of mathematical formulation used in the MILP model in Section “Mathematical Formulation and Solution Procedure.” An industrial case study is then used to explore the capabilities of the model and identify trends, which can be used to aid business decisions (Section Illustrative Example). The mathematical nomenclature can be found at the end of this article.

## Problem Definition

The focus of this work is on long-term multi-site production planning for biopharmaceuticals to minimize the total manufacturing cost and investment whilst satisfying demands. The key features of the problem are discussed below.

### Facility features

Allocation of biopharmaceutical facilities across multiple sites requires an understanding of the different facility features such as scale and capability to manufacture each product as well as any differences in fermentation titres and downstream processing (DSP) yields. The number and size of bioreactors will directly affect a facility's upstream processing (USP) capacity. The same product could be manufactured in two different facilities, with each facility having a different number of bioreactors available; hence, the optimization will select, which facility to use based on cost and capacity requirement. DSP scales will also vary as there may be different sized purification equipment such as chromatography columns or filtration rigs. Depending on how the DSP is set up, it could mean that the time required for purification is different between facilities. For example, if the same amount of material is to be processed by a facility with a smaller filtration device, that particular step will be slower (when compared to a larger filtration unit with greater throughput). There may also be multiple DSP trains to process the material from one harvest, which is common for antibody production with high titres.^22^ Conversely, operators could decide to keep the purification time constant, but change the amount of material processed. These process choices must be correctly captured in the model for there to be realistic manufacturing flexibility. The capability of a facility to manufacture a product will differ, not just owing to logistical aspects, but also strategic. For example, if a future facility is built with perfusion products in mind, then it may not be possible to later manufacture fed-batch products. A CMO, however, may be capable of manufacturing all of the products, but due to licensing and IP issues a company may wish to keep production of certain products in-house. Another key facility feature is the cost of manufacturing a product there. Cost differences are present between in-house and CMO facilities to reflect the extra service cost with CMOs.^21^

### *Fed-batch* *vs*. *perfusion* *culture* *processes*

The USP stages of mammalian cell culture processes typically involve either fed-batch or perfusion culture.^23^ It is also possible to have one or more steps of the seed train as perfusion-mode, and the production cell culture as fed-batch.^24^ Perfusion culture is necessary for labile products such as blood factors and enzymes (e.g., Cerezyme®) and has also been used for certain stable monoclonal antibody (mAb) products (e.g., Remicade®) using retention devices that range from gravity settlers to filtration devices.^23^ Perfusion processes typically offer higher daily volumetric productivities, and hence smaller facility footprints than fed-batch culture strategies.^23^,^24^ However, they are generally more complex to operate, require increased amounts of media, and are susceptible to higher failure rates.^23^,^25^ Newer perfusion retention devices using external tangential flow filters aim to overcome some of these obstacles with the capability to attach to single-use bioreactors combined with lower failure rates and higher productivities.^26^ This has increased interest in the business case for perfusion-based processes and process economic analyses have explored the cost-benefit of perfusion vs. fed-batch processes.^23^,^25^ However, in recent years, fed-batch culture has become the platform choice for most mAbs due to dramatic increases in fed-batch titres combined with ease of operation.^22^,^25^

**Figure 1:**
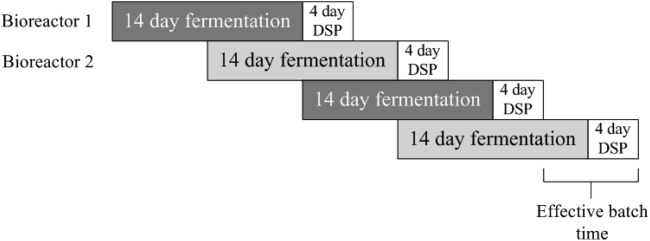
Batch-mode process using two staggered bioreactors.

**Figure 2:**
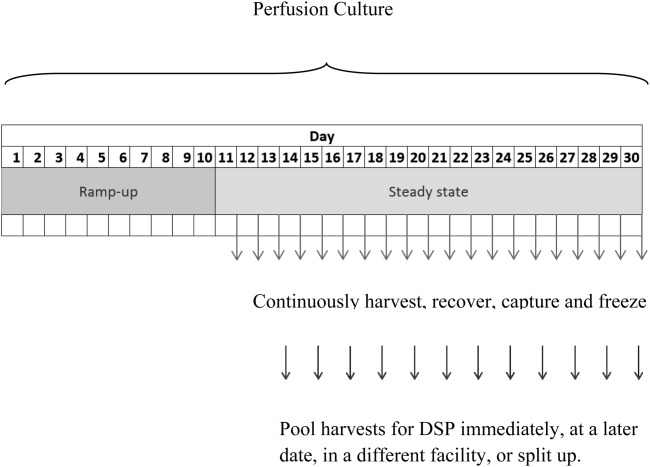
Perfusion mode cell culture.

The USP mode of operation has a direct impact on the scheduling of the subsequent DSP steps. In fed-batch mode (Figure 1), the culture is harvested at the end of the cell culture duration and subsequently purified by a series of DSP steps (e.g., chromatography). In perfusion mode, material is continuously harvested, recovered, captured, and sometimes frozen throughout the fermentation culture. Once enough material has been pooled together, it is purified downstream (Figure 2). There is also a set amount of time required for quality and assurance tests after each harvest before it can be processed downstream. The ramp-up time is the time required for the cell culture to reach a certain cell density, after which steady state is achieved. Material is sometimes harvested during the ramp-up time, but in this work it is assumed to be discarded. The DSP can be carried out immediately or at a later date, either within the same facility or a different one should there be financial incentive. The DSP can only be carried out immediately after harvesting if no quality release testing is required. Perfusion cell cultures usually operate for longer than fed-batch cultures, and as no clean-in-place or steam-in-place can occur during this time, there is a greater risk of contamination.^27^ For perfusion processes, sterility samples are taken every day and viral samples are taken every 2 weeks. Extra testing may be required for longer cell culture durations as the risk of contamination is increased the longer a bioreactor is operating for.

Perfusion processes can, therefore, be modeled as a black box, where DSP directly follows USP, or using a decoupled design. The flexibility in perfusion-mode manufacturing is only apparent if USP and DSP are decoupled from each other. The black box design is simpler and can, therefore, be solved quicker, but the decoupled design allows for more manufacturing flexibility, which could (depending on input parameters) provide a lower overall manufacturing cost and is also a closer representation of reality.

### Key performance indicators

Successful production planning requires consideration of cost factors such as the manufacturing cost, the capital investment required either to build new facilities or retrofit existing ones, as well as inventory costs. The manufacturing cost can be separated into fixed and variable costs. This model assumes the variable cost to consist solely of materials, with costs attributed to labour, depreciation, and facility overheads being assigned to fixed costs. The inventory cost includes the actual warehouse costs as well as the cost attributed to the opportunity lost in selling the product. In addition to costs, customer service levels can be assessed to see how much customer demand is met on time. Insufficient capacity will lead to lower customer service levels. It is also important to determine the facility utilization to avoid idle expenses. Facility utilization may need to be kept within certain targets. If facility utilization is too high, any unplanned downtime could severely affect the customer service level. Underutilization, conversely, may suggest a misplaced investment in capacity. Together, these performance indicators help a manufacturer to assess the viability of a production plan.

## Mathematical Formulation and Solution Procedure

The following section describes the mathematical formulation developed to address the problem domain. The nomenclature can be found at the end of this article. It is important to note that many of the variables have been duplicated for the upstream and downstream parts of the model (e.g., the number of batches produced). To aid with legibility, the superscripts “U” or “D” denote upstream or downstream, respectively. This model uses a discrete time representation, with monthly time resolution. This means, for example, that for an 8 year planning horizon there would be 96 time periods.

### Technical and commercial constraints

#### Production Constraints

In essence, the number of upstream batches produced in time period *t*, for product *p*, in fermentation suite *i*, is denoted by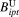 and is equal to the batch rate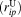 multiplied by the amount of time available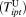. If there is a changeover between products *p'* and *p*,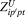 will equal 1, and a campaign changeover time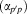 is subtracted from the available time. Depending on whether the product is manufactured using fed-batch or perfusion culture, an additional time is subtracted. For perfusion products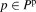 (Eq. (2)), the ramp-up times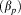 are subtracted when new perfusion cell cultures begin (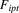 = 1). For fed-batch products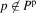 (Eq. (1)), the time required for the first batch (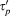) is subtracted so that the effective batch rate can be used from that point onward. For example, if the fed-batch process is like that shown in Figure 1, the first upstream batch would take 14 days, but from that point onward there will be another batch every 7 days. The extra time necessary for the first batch is only required when a new campaign starts (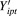 = 1). To compensate for the removal of time for the first batch,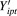 is added to the number of batches. Hence, when a new campaign of a fed-batch product begins,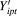 is equal to 1, and the number of batches produced is equal to 1 plus the effective batch rate multiplied by time available minus time required for the first batch.


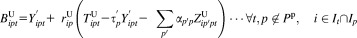
(1)


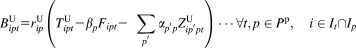
(2)

To ensure only one product is manufactured in a suite at any given time, the binary variables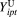 and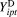, which are equal to 1 if product *p* is manufactured in suite *i* at time *t* for upstream and downstream suites, respectively, are constrained as follows:


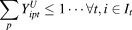
(3)


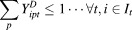
(4)

New upstream campaigns are indicated with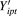 being equal to 1, and this can only occur if there was no production of that product in the previous time period.


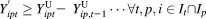
(5)

The number of upstream batches for products using fed-batch mode,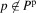, is equal to the number of batches in the purification (assuming there is no pooling or splitting of fermentation volumes):


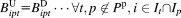
(6)

For perfusion products, the number of downstream batches is simply equal to the batch rate multiplied by the amount of time available:


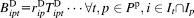
(7)

*Availability Constraints*. In order for production to take place in a facility, it must first be available for use. It may first need to be built, retrofitted, or may even be unavailable for another reason (e.g., being used for another product which is not in the current product portfolio). The variable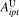 is equal to 1 if facility *i* is available to product *p* at time *t* for upstream production. Variable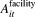 is equal to 1 if facility *i* has been built and is ready to be used at time *t*, and variable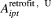 is equal to 1 if facility *i* has been retrofitted for product *p* and is ready to be used at time *t* for upstream production.


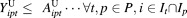
(8)


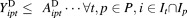
(9)


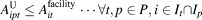
(10)


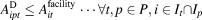
(11)


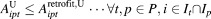
(12)


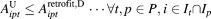
(13)

The availabilities for building a facility or retrofitting (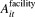,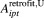) are linked to the investment constraints which follow.

#### Investment Constraints

Before a facility can be used, there must first be investment into the construction of that facility. The facility is not available before the time is takes to construct it (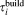). Construction starts as soon as investment is made. The variable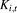 is equal to 1 if capital is invested at time *t*.


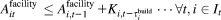
(14)

In order for a product to be manufactured in a facility, any relevant retrofitting must be carried out.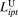 is equal to 1 if facility *i* has been retrofitted for product *p* at time *t*. The investment for retrofitting must be spent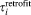 time periods before the facility becomes available for that product.


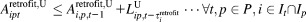
(15)


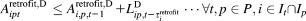
(16)

The model also includes any licence fees and start-up costs, and this is indicated via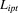. There is no differentiation between upstream and downstream here as a licence is assumed to be required per facility, not per suite. If there are special licences or costs that are applicable to suites rather than facilities, then they can be incorporated into the retrofitting costs.


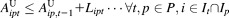
(17)


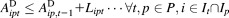
(18)

#### Fixed Cost Constraints

A simplified fixed cost model is used to calculate the annual fixed cost in each facility. Generally, the products would have different fixed costs, and thus, the annual fixed cost would be the maximum of the fixed costs of the products produced in that year. If no product is manufactured in a given year, then there is still a fixed cost applied because the facility still needs to be maintained under Good Manufacturing Practice (GMP) conditions. Upstream and downstream suite use (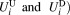 is separated so that fixed costs can be attributed individually. If a suite has never been used over the planning horizon (e.g., if it had never been built, or if no product was ever allocated to it), then no fixed costs need to be applied for that suite. Also note that only the facilities which are owned (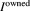) need to be subjected to fixed costs. This is achieved in the objective function where the cost is applied.


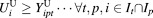
(19)


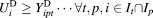
(20)

#### Timing Constraints

To tighten the optimization's search for an integer number of batches, a minimum processing time can be enforced. The maximum utilization time in any given month,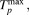 is usually just equal to 30 days, but in some cases this can be adjusted to tighten the optimization.


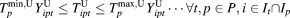
(21)


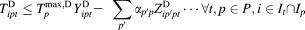
(22)

Changeovers occur when there is a product switch within the same facility. In the following equations,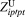 is equal to 1 when there is a changeover from product *p′* to *p*. If there is an idle period, this model will assume that the changeover will take place in the idle period and thus will not subtract from available production time.


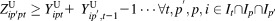
(23)


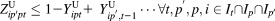
(24)


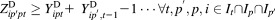
(25)

For perfusion products, new cell cultures start (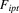 = 1) when a new campaign starts:


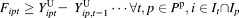
(26)

As perfusion cell cultures have a fixed length (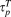), it is necessary to ensure that a new cell culture is started once the previous one has finished.


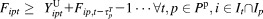
(27)

The following constraint ensures that the perfusion campaign is run for its entire length, and that each day in the month is also used. This last point is important as once a perfusion process has started, it should be run continuously, and thus there cannot be idle days in the middle of the cell culture. The cell culture's duration in days and time periods are represented by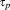 and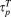, respectively. Thus, if a new 150 day cell culture is started,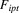 will be equal to 1, and so the equation forces the total time used during the cell culture to be equal to 150 days. Note that although the equation does not explicitly restrict the total time, it is limited in the timing constraint from earlier (Eq. (21)).


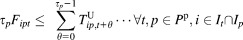
(28)

The following constraint is needed to prevent the situation where perfusion campaigns are started near the end of the planning horizon, without enough time to finish. It also solves a problem, where *F_ipt_* can potentially be equal to 1 even if it is not the beginning of a new perfusion campaign (this can happen if the model wishes to add downtime to lower the cost or meet a constraint).


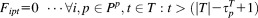
(29)

#### Inventory Constraints

The constraint shown in Eq. (30) states that the inventory level for the fermentation product (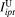) is equal to its previous level plus any material produced in subsequent batches (taking into consideration quality checks of duration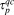), minus any material which is used for purification (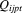). The amount of material produced in one time period is equal to the output per batch (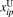) multiplied by the number of batches, and is adjusted using a rejection coefficient (*R*). So if 5% of material is rejected, then 95% of the material from the batches can enter the inventory.


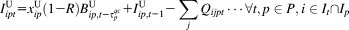
(30)

The flow of material from the fermentation suite, *i,* to the purification suite, *j,* is characterised by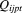. Aforementioned, the lot size for the purification train is fixed for each product, and this is enforced by the following constraint:


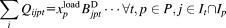
(31)where *i* is the fermentation suite and *j* is the purification suite. Equation (31) states that the total flow of material in a given time period from all the fermentation suites to the current purification suite must equal an integer number of batches multiplied by the batch lot size. This constraint means that material can be pooled from different fermentation suites and processed as one batch in a DSP suite. This is an assumption in the model and should be adapted if pooling is not allowed.

The downstream inventory level of product *p* in time period *t* in facility *i* is equal to the amount produced (taking into consideration production losses) plus the previous month's inventory level, minus any amount of material sold (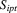) or wasted (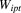). The amount sold is limited by demand (Eq. (39)). Assuming all material here is used, the amount produced is simply equal to the output per batch (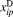) multiplied by the number of batches (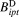).


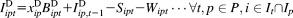
(32)

In any given time period, the model will try to maintain the strategic inventory level (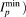 by calculating the gap between the inventory level and the target (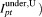, and then penalizing this variable in the objective function.


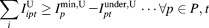
(33)


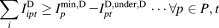
(34)

#### Utilization Constraints

There are maximum utilization targets for in-house facilities, and thus constraints need to be put into place to accomplish this. For every in-house facility and each year, the following equations restrict the total time used for each product in each month of the year to be below the maximum allowed. Therefore, if the maximum desired facility utilization is 75%,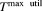 can be set to 270 days. The model applies the same utilization target to both upstream and downstream suites.


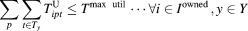
(35)


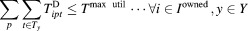
(36)

#### Shelf-Life Constraints

The products have a limited shelf-life (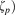, and so a constraint needs to be introduced (Eq. (38)) to ensure that the product is sold before its shelf-life expires. Also, the intermediate product from the upstream process must be purified before it expires (Eq. (37)).


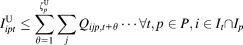
(37)


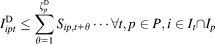
(38)

#### Sales Constraints

To allow for feasible solutions in situations where demand cannot be met, a backlog variable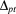 is introduced. This variable is then penalized in the objective function so as to ensure as much demand is met as possible. Some products (notably those which use perfusion) require quality checks before being passed to purification, and thus this time must be considered when meeting the demand. If the demand for a certain product is in month eight, but it takes one month to perform the quality checks, then the material must be ready by month seven, ensured by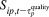.


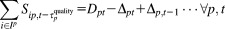
(39)

### Objective function

The discount factor is calculated as:


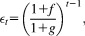
(40)where *f* is the rate of inflation and *g* is the interest rate.

The individual costs have been broken down as follows:


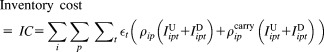
(41)


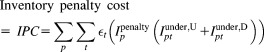
(42)


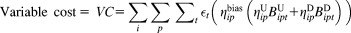
(43)


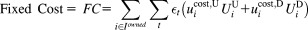
(44)


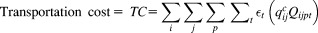
(45)


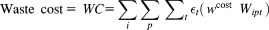
(46)


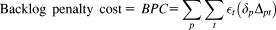
(47)


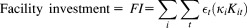
(48)


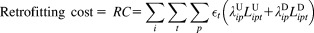
(49)


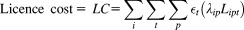
(50)

The total cost consists of all the above costs summed together, and finally Eq. 1-51 form the MILP problem to be optimized.


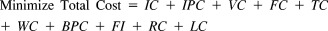
(51)

### *Optimi*z*ation* *strategies*

To obtain a good solution within reasonable time becomes increasingly more difficult as the number of products, facilities or time periods being captured rises. To achieve better solutions, a rolling time horizon was used, whereby a smaller optimization problem was run first, and part of the solution to this subproblem was used to initiate the subsequent larger problem. For example, if the capacity plan was for 8 years, the first subproblem could be a 4-year plan, and once this has solved the second subproblem could be 5 years in length, but with the binary variables in the first year fixed to the solution from the previous subproblem. The next subproblem would be 6 years in length, with the first 2 years fixed from previous solutions, and the process continues until the full 8 years has been captured. Although this approach is unlikely to find the true optimum (as optimality gaps are accumulated for each subproblem), for the example investigated in this work it can provide better solutions than the full scale optimization under finite time. It should be stressed that given an unlimited amount of time, the full scale mode will always provide the best solution. Table1 shows a rolling time horizon where only 4 years are actually optimized in any given subproblem, with the time horizon expanding by 1 year each time, fixing the binary variables of earlier years using the solution from the previous subproblem. The rolling horizon approach implemented in this work avoids infeasible situations by allowing backlogs to accumulate if demands in future years are greater than the model was previously able to detect in the subproblems. Backlogs are penalized in the objective function, hence inferior solutions could arise. However, in the base case presented here, the rolling time horizon approach performed better than the full model in finite time.

**Table 1:** Illustration of a Rolling Time Horizon.

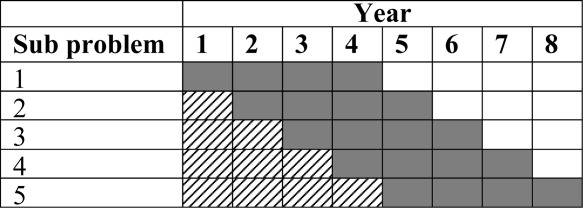

Dark-grey boxes show years where optimization takes place, white boxes where no optimization occurs, and diagonally shaded boxes where the binary variables are fixed from the previous solution.

## Illustrative Example

### Input data

This framework is tested on a case study of a generic portfolio of four drugs and four facilities, key details of which are listed in Tables5. Representative data for this case study were derived from literature sources (e.g. 22,23,28), as well as through discussions with industrial practitioners involved in fed-batch and perfusion processes and production planning. The four facilities consist of two in-house facilities, one contract manufacturer, and one facility that can be built in the future if required. The four products are in differing stages of clinical trials, but the demands modeled here are for when the products reach the consumer market. Hence, the points where demands start in Table4 differ according to how close the product is to market penetration. The time horizon for this case study is 8 years.

**Table 2:** Process Data for Drugs in Case Study

		Product			
		*p*_1_	*p*_2_	*p*_3_	*p*_4_
**Process Data**					
USP					
Fermentation mode		Perfusion	Perfusion	Perfusion	Fed-batch
Cell culture duration (days)		150	60	28	14
Ramp-up time (days)		10	10	10	N/A
Harvest (AU*/day)		14.3	37.8	4.8	N/A
Shelf-life (months)		24	24	24	N/A
QC/QA time (days)		30	30	4	N/A
*DSP*					
Lot size (AU*)		450	1,000	720	105,000
Duration (days)		1.5	1.5	4	4
Shelf-life (months)		24	24	24	24
**Cost Data**					
USP					
Variable cost (RMU†/AU*)		0.05	0.05	0.225	0.018
Fixed cost (RMU†/year)		65	65	65	65
*DSP*					
Variable cost (RMU†/AU*)		0.002	0.002	9,000	100
Fixed cost (RMU†/year)		48	48	48	48
Sales price (RMU†/AU*)		6	6	27	0.1

* Arbitrary units.

† Relative monetary units.

The quality control/quality assurance (QC/QA) time shown in Table2 is only applicable to perfusion processes, whereby the intermediate frozen material from fermentation is checked prior to purification. This can lead to a substantial lag between material being produced in the fermentation step, and it being able to be purified and thus meet demand, hence is included in the model.

Not all products can be manufactured in every facility, and for those combinations which are allowed there may be a one-off retrofitting cost associated with initial production. For example, for strategic reasons a company may wish to keep the production of one of their products to in-house facilities only, and thus CMOs would not be available for its manufacturing. To use in-house facilities, however, retrofitting is required, which must be taken into account during the optimization. Other products may not be able to be manufactured in a facility simply because the correct equipment is unavailable and retrofitting may be infeasible. Table3 shows the production relationships between the products and facilities in this case study, and also states which combinations require retrofitting.

**Table 3:** Facility Manufacturing Capabilities

Facility	Product			
	*P*_1_	*P*_2_	*P*_3_	*P*_4_
*i*_1_	Y^*^	Y^*^	Y^*^	Y ^*^
*i*_2_	Y	Y	*N*	*N*
CMO	*N*	*N*	Y	Y
Future	Y	Y	*N*	Y

Note: Product can (Y) or cannot (N) be produced in facility. Retrofitting requirement denoted by ^*^. Facilities *i*_1_, *i*_2_ and the future facility are owned.

**Table 4:** Product Demand and Strategic Inventory Levels (arbitrary units, ×10^3^)

	Year								Strategic Inventory	
	1	2	3	4	5	6	7	8	USP	DSP
*p*_1_	0	20.2	20.3	20.5	21.4	27.2	28.3	29.9	8.6	26.4
*p*_2_	0	0	1.1	3.2	5.3	7.4	9.5	11.5	22.7	19.2
*p*_3_	0	0	0	0.4	0.4	0.4	0.44	0.48	2.2	0.2
*p*_4_	0	0	0	0	2,500	2,750	3,030	3,330	N/A	1,900

**Table 5:** Initial Start-Up Costs (including Retrofitting, CMO Negotiation Fees, and Licences) in Relative Monetary Units.

			Product			
			*p*_1_	*p*_2_	*p*_3_	*p*_4_
USP	*i*_1_		32.5	32.5	32.5	32.5
	*i*_2_		0	0	–	–
	CMO		–	–	7	7
	Future		10	10	–	10
				
DSP	*i*_1_		87.5	87.5	32.5	32.5
	*I*_2_		0	0	–	–
	CMO		–	–	7	7
	Future		10	10	–	10

Table4 shows what the desired inventory levels for the intermediate frozen material and final DSP products are, and it is assumed that these levels remain constant throughout the 8 years of capacity planning. In reality, these figures would probably change as they are influenced by annual demand, and thus as demand increases over the years so would the strategic inventory level.

Once a product has shown promise and the company wishes to expand to commercial manufacturing, a biologic licence application and prescription drug user fee needs to be applied for (this can total just over $2M).^29^ Each time a product is manufactured in a new facility, a licence application needs to be submitted, and thus the model will try to minimize the number of licences applied for and keep production limited to one facility if possible. Table5 shows the different costs associated with starting production in a particular facility for a certain product. These costs include the licence costs mentioned previously and retrofitting costs (new equipment and facility utilities).

There are also changeover times between the products, as listed in Table6, which are important to model as when the process mode changes from perfusion to fed-batch, there can be large amounts of downtime due to swapping large unit operations which cannot be shared.

**Table 6:** Changeover Times between Products.

	Product ( 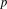 )				
		p_1_	p_2_	p_3_	p_4_
Product ( 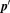 )	*p*_1_	7	7	7	14
	*p*_2_	7	7	7	14
	*p*_3_	7	7	7	14
	*p*_4_	14	14	14	7

The units are in days, and represent the time taken to change from product *p′* to *p*.

### Computational results

**Figure 3:**
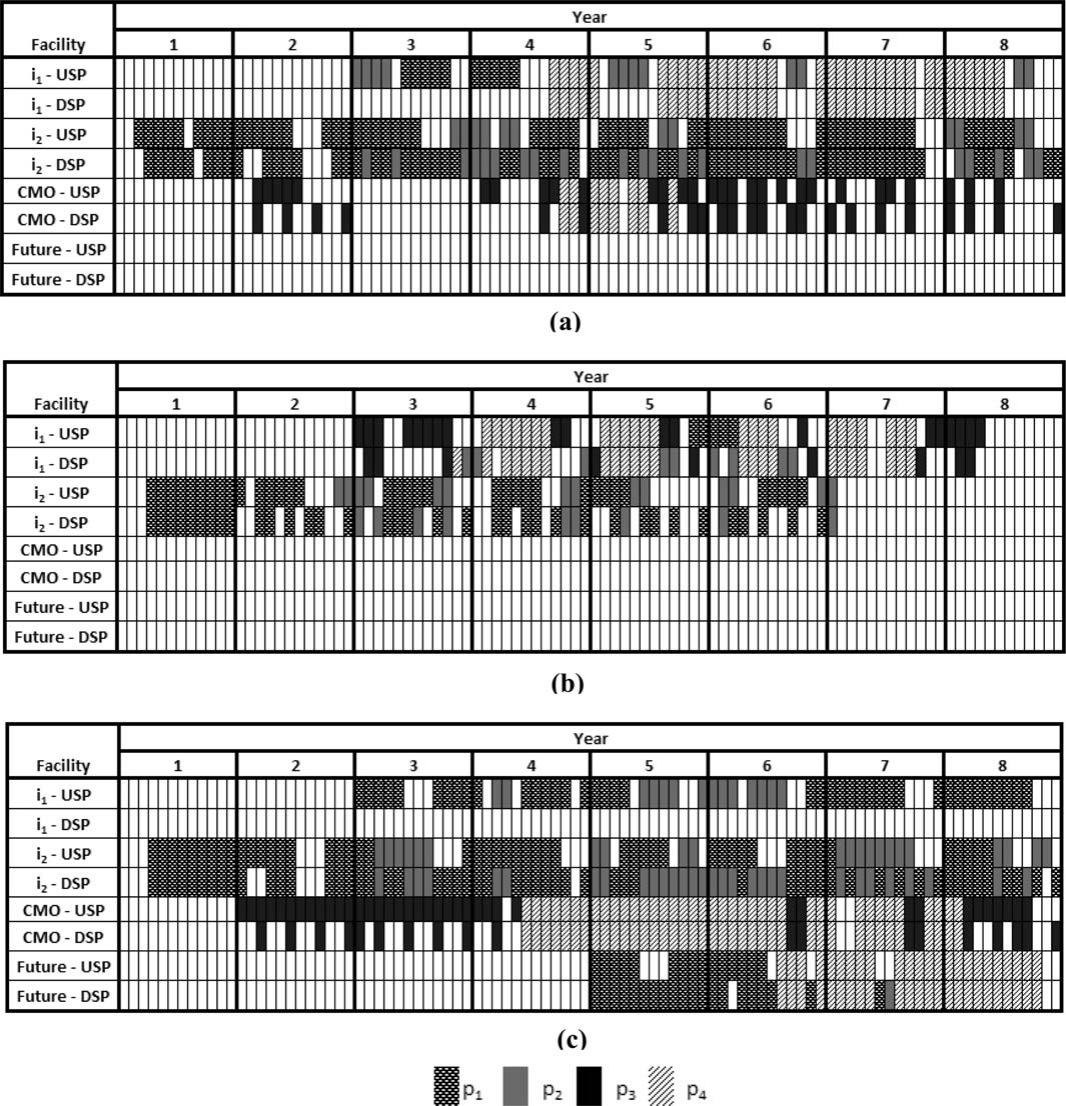
Manufacturing schedule for the base case (a), −50% demand (b) and +50% demand scenario (c).

The model predicted the production plan of the four products across four available facilities. The results in Figure 3 show the plan over 8 years for different demand scenarios. The base case requires the use of a CMO to meet the demand for *p*_3_ and excess demand for *p*_4_. Note that the manufacturing of a product is kept within one facility if possible so as to minimize licence fees. It is clear to see that when the demand is low, all production can be met in-house and without further expansion. Higher demands require almost full use of all the facilities available, and expansion to a CMO and new facility. Despite not being shown here, the market demands for all the products were met in full for almost all scenarios (100% customer service level). Only in the last year of the +50% demand case was there a small backlog for *p*_3_ and *p*_4_ (customer service level of 95%). Therefore, from a strategic viewpoint, the scenario with higher demand looks less robust as the facilities are heavily utilized and there are already small backlogs accumulating. There is very little margin for error should there be a contamination or failed batches, thus extra capacity would be desirable.

**Figure 4:**
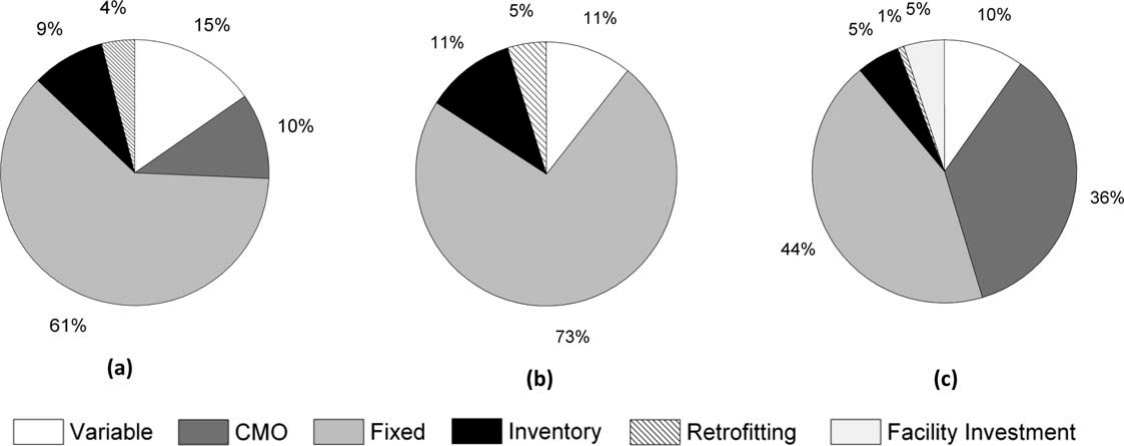
Cost breakdown for different demand cases.Base case is shown in (a), 50% decrease in demand in (b) and 50% increase in demand in (c).

A cost breakdown for the three demand cases shown in Figure 4 was conducted and shows a clear increase in cost attributed to CMO activity in the higher demand case (Figure 4c). For in-house production, the ratio between variable to fixed costs ranges from approximately 1:7 (low demand) to 1:4 (high demand). This range is justifiable as the demand increases, so too will the variable costs, whilst the fixed costs will remain unchanged. It should be noted that for this particular case study, once production in a facility has started, annual fixed costs will be applied to that facility from that point onward, because most activities included in the fixed costs (such as labour, facility maintenance, and cleaning) will be on-going even if there is an idle year. The higher demand case also shows that 5% of the total cost comes from the investment required to build the new facility.

**Figure 5:**
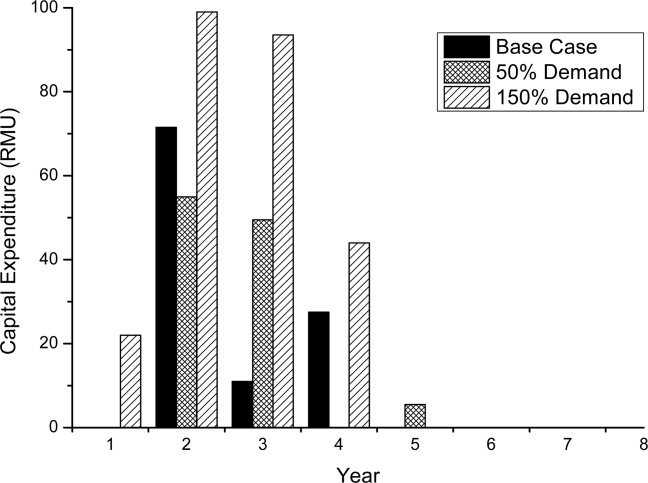
Capital expenditure profiles for the base case, 50% demand, and 150% demand.

Capital expenditure information for all three demand cases is shown in Figure 5, and correlates to the retrofitting and facility investment costs in Figure 4. The capital expenditures for the reduced demand case and base case are only that of retrofitting. For the higher demand scenario, a new facility is required, and the cost of building the facility is spread out over 4 years, hence the expenditure between years 1–4. Retrofitting costs are minimized by the model attempting to keep production within one facility if possible.

**Figure 6:**
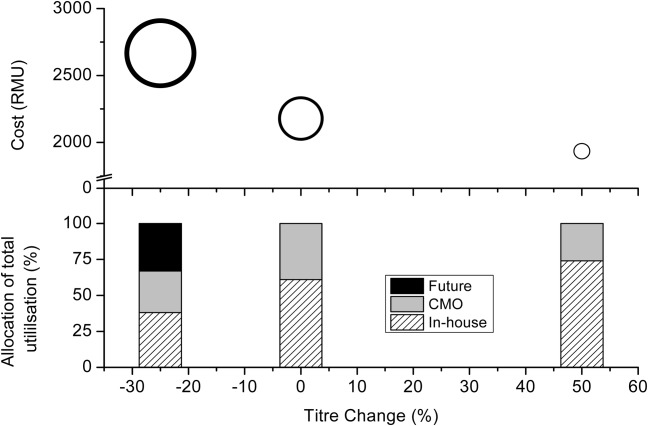
Cost and utilization vs. titre variance.The size of the bubbles represent the amount of extra capacity required to meet the demand. This extracapacity could be sourced from a CMO, or a future facility. The utilization percentage used for this figure relates to the USP only.

Another scenario that may occur is variability in titres for certain products. Process parameters for products in early stages of development are not as well known as process parameters for commercial products or products in late stage development. Also, when approaching a CMO they may have superior technologies which can boost titres. Scenarios were carried out to see how varying the titres for *p*_3_ and *p*_4_ by a 25% reduction and 50% increase could affect the capacity requirement (Figure 6). These two products were chosen because less was known about their manufacturing processes as they were in early clinical trials. Products *p*_1_ and *p*_2_, conversely, were nearing the end of their trials; hence, process parameters are known with greater certainty. The reason the titre is varied from −25 to +50% is based on the assumption that if there were to be titre changes/fluctuations, it is more likely to be in the positive direction due to ongoing research, improving cell lines or process design. There is still the risk, however, that the process may not scale well, and hence lower titres are also examined. Titre variations of ±20% are not uncommon when scaling up a process.^30^ For example, a lower titre cell line may be selected if it generates fewer host cell impurities or demonstrates more consistent behavior. When the titre is lower than expected, a much larger proportion of external capacity is required, both in the form of a CMO and through building a new facility. The choice of whether to go to a CMO or build a future facility is mainly influenced by cost and is discussed later on. Note although, that under the base case conditions a CMO is the preferred choice as there is much less capital investment required, and the fixed overheads (which are the dominant costs for in-house production) are no longer applied in the same way as for in-house facilities. The CMO would still pass on its fixed costs to its customers, but if manufacturing does not span an entire year and the CMO has other clients, this will amount to less than would have otherwise been spent in-house. A CMO alone would not have been enough to meet demand for the reduced titre scenario; hence, the future facility was required. With higher titres, the model pushes for a greater proportion of in-house manufacturing (75%) as there is now unused capacity in the existing facilities.

**Figure 7:**
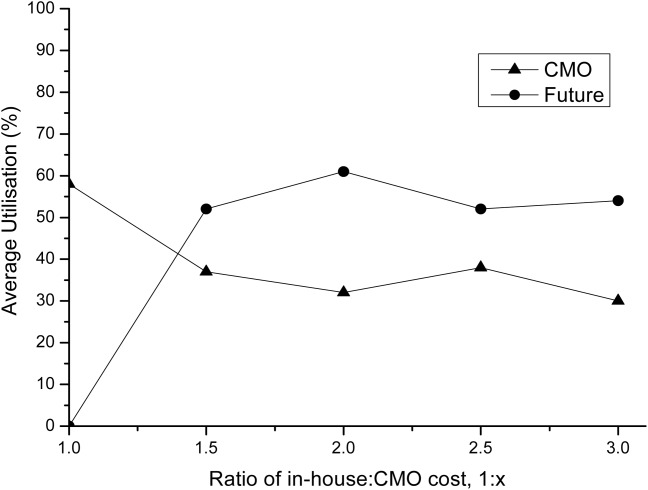
Utilization of CMO and future facility vs. ratio of in-house to CMO manufacturing cost.The utilization percentages displayed here are for the USP production and are the average monthly utilization from the moment the facility is used to the end of the 8 year capacity plan.

This case study includes a CMO in the list of available facilities, and as such the costs of production there will be different to the in-house production costs shown in Table2. The cost of production in a CMO can be up to three times greater than in-house manufacturing, depending on the scale of production.^4^,^22^,^31^ In the base case, we have stated that the CMO costs are 50% higher. However, this is only an assumption, and hence the model was used to show what would happen to the capacity plan if the CMO costs were to change. CMOs are naturally more expensive than in-house production as they not only need to cover their costs but also charge commission. The extra amount that is paid will be dependent on the CMO's experience, location and technology it has to offer. Given that the CMO costs in this particular case study are uncertain, an analysis was conducted to see how much more expensive the CMO had to become before it became cheaper to build a new facility and produce in-house. Figure 7 shows that once the CMO becomes 50% more expensive than in-house production, a future facility provides alternative means of meeting market demand at lower costs. The utilization of the CMO decreases as the cost of the CMO increases, but it never reaches 0% (even at 10 times the cost of in-house production) because there is simply not enough capacity in the existing facilities for the fermentation of *p*_3_ (which is being produced in the CMO). On top of this, the future facility cannot produce *p*_3_, hence the fall in utilization for the CMO is not as large as one would initially expect.

**Figure 8:**
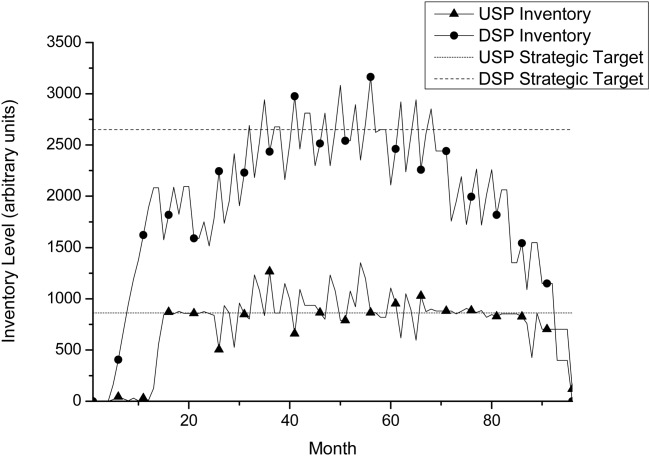
Inventory profile for *p*_1_, including both USP and DSP levels.The optimization attempts to maintain strategic levels, but it will always place more importance on meeting demand first.

Inventory profiles are useful to see whether the results are what one would expect as they clearly show whether the targets are being met and if there is a lot of variation. Figure 8 shows the inventory profile for a perfusion-mode process, and thus includes the upstream inventory level as well as the downstream level. The figure also includes the strategic inventory levels that should be maintained throughout the capacity plan. As noted before, the levels may in reality change over time, but this model assumes them to remain constant. The figure quickly demonstrates to a manager that the correct inventory levels are being maintained in the middle of the plan, but near the end the levels tend to drift downward toward zero. This is actually owing to the fact that the penalty applied for being under the strategic level in the objective function is applied on a monthly basis, and thus near the end there are fewer months available to penalize the shortfall, hence being under the strategic level no longer has such a detrimental effect on the objective function. It, therefore, becomes cheaper to have less product in storage.

**Figure 9:**
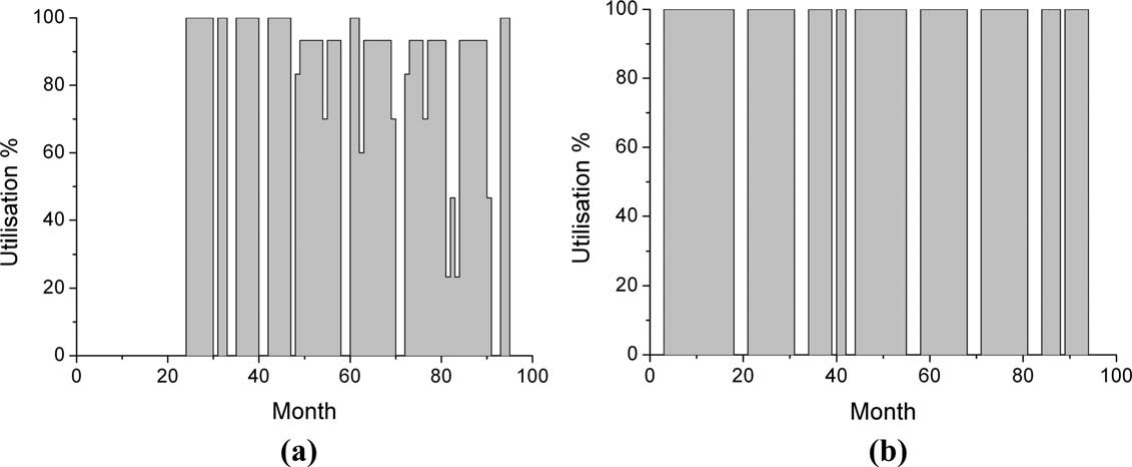
Monthly utilization charts for in-house facilities *i*_1_ (a) and *i*_2_ (b) for the base case.The percentages are of all products aggregated together for the fermentation (USP). Note that *i*_1_ cannot be used for the first 2 years as during that time another product (not modeled here) has been designated to it.

Utilization graphs can also be used to detect if extra capacity could be directed toward an existing facility with low utilization, or whether a facility is deemed to be utilized too much and hence raises risk concerns should there be any unplanned downtime. Figure 9 shows how facility *i*_2_ is almost at maximum capacity, with only small breaks in production. The breaks in production are actually there on purpose as there is an utilization cap of 75%. This provides leeway should problems with failed batches occur. Facility *i*_1_ still has some available capacity, but not enough to meet all demand, hence why the CMO is used in the base case.

### Computational statistics

The optimization was performed on an Intel Xeon W3565 Quad-core 3.2GHz processor, with 6 GB random access memory (RAM) running Microsoft Windows XP 64-bit. The framework presented in this article uses the CPLEX 12.5.1 solver^32^ within the General Algebraic Modeling System (GAMS) 24.1.3^33^ to solve the MILP problem, and outputs the solution to Microsoft Excel for analysis using Visual Basic for Applications. All optimizations (full-scale and rolling time horizon subproblems) were completed to within 5% optimality.

Although, the case study outlined previously is relatively small, in that it only consists of four products and four facilities (each with upstream and downstream suites), the problem itself is computationally difficult to solve. Table7 shows how the number of variables and constraints in the model increases substantially with increasing numbers of products, facilities, and time periods. It should be noted that these numbers would fluctuate depending on the individual case. For example, if a product cannot be manufactured in a certain facility, then a set of constraints and variables would be eliminated. The statistics for the case study presented in this article are represented by the bold highlighted case in Table8. It is clear that the model could become considerably larger in size if just a few extra products or facilities were added to the case study. Hence, as the problem size increases it becomes more critical to adopt solution strategies that make the problem tractable, such as a rolling time horizon.

**Table 7:** Model Statistics for Various Numbers of Products (*p*), Facilities (*i*), and Time Periods (*t*).

Case	Constraints	Continuous Variables	Discrete Variables
2*p*, 2*i*, 48*t*	6,605	4,719	1,148
**4*p*, 4*i*, 96*t***	**33,183**	**25,037**	**5,184**
8*p*, 8*i*, 192*t*	426,567	350,349	45,696

The case study presented in this paper is highlighted in bold.

**Table 8:** Comparison between the Computational Results for the Full Scale Problem and the Rolling Time Horizon.

Case	Obj. Func. (min)	Optimality Gap	CPU sec
Full scale	3,389	13.1%	10,800
Rolling 3/1	3,467	15.1%	140
Rolling 4/1	3,327	11.5%	1,484

Note: In the full scale model all 8 years were planned for simultaneously. In the rolling time horizon approach, either 3 or 4 years were being optimized whilst expanding the horizon by 1 year for each subproblem. The time reported for the rolling horizon approach is the sum of all subproblems. The optimality gaps shown for the rolling time horizons are calculated based on the best bound from the full scale model. Each subproblem was optimized to within 5% optimality.

Table8 shows a clear improvement in using a rolling time horizon, both in terms of obtaining a better optimal solution and also a reduction in CPU time. Obviously, by solving multiple subproblems (each to a 5% optimality gap), the best bound in the final subproblem will have accumulated a divergence from the full scale problem, hence for comparison the best bound for the full scale problem is used for calculating all the optimality gaps. The 3/1 rolling horizon approach seems to offer the most in terms of computational speed, whereas the 4/1 approach finds a better solution but at the cost of extra computational effort. Compared to an optimization of 3 h with the full scale model, the 4/1 rolling horizon provides a better solution within much less time.

## Concluding Remarks

This article has demonstrated how production plans for fed-batch and perfusion bioprocesses can be optimized using mathematical modeling by incorporating various costs and time constraints, including sequence-dependent changeover times. Both the upstream and downstream processes have been incorporated into the model, and decoupled by the use of an intermediate storage step, which allows greater flexibility for perfusion processes. The results demonstrate how capacity plans can be quickly determined for various scenarios, aiding the manufacturer in deciding when to consider outsourcing production, and the capital expenditure likely to be required.

The solutions acquired using this framework were improved through a rolling time horizon solution procedure, and the CPU time required was also substantially reduced. Future work will include incorporating features to maintain strategic inventory levels throughout the time horizon, addressing multiple objectives, and reducing the optimality gap even further by appropriate model reformulations.

## Notation

### Sets

=facilities
=owned facilities
=facilities which can produce product *p*
=facilities which are available in period *t*
=products
=perfusion products
=products which can be produced in facility *i*
=time periods
=time periods which are in year *y*
=years.

### Scalars

=time horizon(days)
=rejection coefficient (%)
=waste cost
=retrofitting time

### Parameters

=changeover time (days)
=ramp-up time (perfusion only)
=backlog penalty cost
=discount factor
=upstream product shelf-life
=downstream product shelf-life
=upstream product batch cost
=downstream product batch cost
=facility investment cost
=licence fees
=retrofitting cost for upstream product
=retrofitting cost for downstream product
=storage cost
=carry of inventory cost
=duration of first batch of a fed-batch process (days)
=perfusion culture duration (days)
=perfusion culture duration (time periods)
=time taken to build facility
=time taken to retrofit facility
=product selling price
=product demand
=penalty applied when strategic inventory level is not met
=strategic inventory level
=strategic inventory level
=cost to transport intermediate material from facility *i* to *j*
=USP batch rate (batches/day)
=DSP batch rate (batches/day)
=maximum production time available within time period
=maximum production time available within time period
=upstream fixed cost
=downstream fixed cost
=downstream lot sizes
=upstream batch output
=downstream batch output

### Binary Variables

=1 if there is a new perfusion culture
=1 if product *p* is manufactured in period *t* in facility *i* (upstream)
=1 if product *p* is manufactured in period *t* in facility *i* (downstream)

### Integer Variables

=number of upstream batches
=number of downstream batches

### Positive Variables

=1 if upstream suite is available
=1 if downstream suite is available
=1 if upstream suite has been retrofitted
=1 if downstream suite has been retrofitted
=1 if facility has been built
=upstream inventory level
=downstream inventory level
=upstream inventory amount under strategic level
=downstream inventory amount under strategic level
=1 if investment for facility *i* took place in period *t*
=1 if retrofitting for facility *i* took place in period *t*
=1 if retrofitting for facility i took place in period t
=flow of material from fermentation to purification
=sales.
=production time used
=waste amount
=1 if new campaign starts
=1 if upstream changeover occurs from p′ → p
=1 if downstream changeover occurs from p′ → p
=demand not met
=total cost (to be minimized)

## Abbreviations

facility (alias)
product (alias)
time period (alias)
year.
